# European clinical guidelines for Tourette syndrome and other tic disorders—version 2.0. Part II: psychological interventions

**DOI:** 10.1007/s00787-021-01845-z

**Published:** 2021-07-27

**Authors:** Per Andrén, Ewgeni Jakubovski, Tara L. Murphy, Katrin Woitecki, Zsanett Tarnok, Sharon Zimmerman-Brenner, Jolande van de Griendt, Nanette Mol Debes, Paula Viefhaus, Sally Robinson, Veit Roessner, Christos Ganos, Natalia Szejko, Kirsten R. Müller-Vahl, Danielle Cath, Andreas Hartmann, Cara Verdellen

**Affiliations:** 1 Centre for Psychiatry Research, Department of Clinical Neuroscience, Karolinska Institutet, and Stockholm Health Care Services, Region Stockholm, Gävlegatan 22, 113 30 Stockholm, Sweden; 2 Department of Psychiatry, Social Psychiatry and Psychotherapy, Hannover Medical School, Carl-Neuberg-Straße 1, 30625 Hannover, Germany; 3 Tic Disorder Clinic, Great Ormond Street Hospital NHS Foundation Trust, London, WC1 3JH UK; 4 School of Child and Adolescent Cognitive Behavior Therapy (AKiP), University Hospital, Cologne, Germany; 5 Vadaskert Child and Adolescent Psychiatry, Budapest, Hungary; 6 Baruch Ivcher School of Psychology, The Interdisciplinary Center (IDC) Herzliya, Herzliya, Israel; 7 TicXperts, Heteren, The Netherlands; 8 Department of Pediatrics, Herlev University Hospital, Herlev, Denmark; 9 Tic and Neurodevelopmental Movement Service (TANDeM), Children’s Neurosciences, Evelina London Children’s Hospital, Guy’s and St Thomas’ NHS Foundation Trust, London, UK; 10 Department of Child and Adolescent Psychiatry, TU Dresden, Dresden, Germany; 11 Department of Neurology, Charité University Medicine Berlin, Berlin, Germany; 12 Department of Neurology, Medical University of Warsaw, Warsaw, Poland; 13 Department of Bioethics, Medical University of Warsaw, Warsaw, Poland; 14 Division of Neurocritical Care and Emergency Neurology, Department of Neurology, Yale School of Medicine, New Haven, USA; 15 Department of Psychiatry, University Medical Center Groningen, Rijks Universiteit Groningen, GGZ Drenthe Mental Health Institution, Assen, The Netherlands; 16 Department of Neurology, Hôpital de La Pitié-Salpêtrière, 75013 Paris, France; 17 PsyQ Nijmegen, Outpatient Treatment Center, Parnassia Group, Den Haag, The Netherlands

**Keywords:** Tourette syndrome, Tic disorders, Treatment guidelines, Behavior therapy, Comprehensive behavioral intervention for tics, Habit reversal training, Exposure and response prevention

## Abstract

Part II of the European clinical guidelines for Tourette syndrome and other tic disorders (ECAP journal, 2011) provides updated information and recommendations for psychological interventions for individuals with tic disorders, created by a working group of the European Society for the Study of Tourette Syndrome (ESSTS). A systematic literature search was conducted to obtain original studies of psychological interventions for tic disorders, published since the initial European clinical guidelines were issued. Relevant studies were identified using computerized searches of the MEDLINE and PsycINFO databases for the years 2011–2019 and a manual search for the years 2019–2021. Based on clinical consensus, psychoeducation is recommended as an initial intervention regardless of symptom severity. According to a systematic literature search, most evidence was found for *Habit Reversal Training* (HRT), primarily the expanded package *Comprehensive Behavioral Intervention for Tics* (CBIT). Evidence was also found for *Exposure and Response Prevention* (ERP), but to a lesser degree of certainty than HRT/CBIT due to fewer studies. Currently, cognitive interventions and third-wave interventions are not recommended as stand-alone treatments for tic disorders. Several novel treatment delivery formats are currently being evaluated, of which videoconference delivery of HRT/CBIT has the most evidence to date. To summarize, when psychoeducation alone is insufficient, both HRT/CBIT and ERP are recommended as first-line interventions for tic disorders. As part of the development of the clinical guidelines, a survey is reported from ESSTS members and other tic disorder experts on preference, use and availability of psychological interventions for tic disorders.

**Supplementary Information:**

The online version contains supplementary material available at 10.1007/s00787-021-01845-z.

## Introduction

Tic disorders are neurodevelopmental disorders characterized by recurrent motor and/or vocal tics. Tics can be transient, as represented by the diagnosis Provisional Tic Disorder in the 5th edition of the Diagnostic and Statistical Manual of Mental Disorders (DSM-5) [1, 2], or they can persist for over a year as a chronic condition, described in the DSM-5 as Tourette’s Disorder (from now on referred to as Tourette syndrome [TS]) and Persistent (Chronic) Motor or Vocal Tic Disorder [CTD]. Typically, tics have an onset between 4 and 6 years of age, are at their worst between 10 and 12 years, and decrease naturally during adolescence and early adulthood [3]. Psychiatric comorbidities are common among individuals with TS/CTD and tend to persist through the life course [4]. For patients who seek TS/CTD-specific treatment, psychological and medical interventions are available.

All parts of the European clinical guidelines for TS and other tic disorders have been created by a working group of the European Society for the Study of Tourette Syndrome (ESSTS). The initial European clinical guidelines were published in 2011 and provided diagnostic and treatment recommendations based on a literature search of clinical trials and case studies up to that point [5, 6]. The current paper is an updated version of part III (now referred to as part II) of these guidelines, focusing on new clinical trials of psychological interventions for tic disorders published after 2011. In addition, early evidence of newly developed psychological interventions for tic disorders are described, including different modalities of treatment delivery, third-wave interventions, as well as overall future directions. Furthermore, results from a survey among ESSTS members and tic disorder experts on preference, use and availability of psychological interventions is reported on. The term TS is used throughout these guidelines for both TS, CTD and Provisional Tic Disorder. Only if there are substantial differences between the tic disorders, a more specific term is used.

## Method

For the current update of part II of the European clinical guidelines, a literature search was performed. The aim was to identify relevant research on the efficacy and effectiveness of psychological interventions for TS, as well as adaptations of interventions when comorbid psychiatric conditions are present, published between January 2011 and June 2019. The databases MEDLINE and PsycINFO at Ovid were searched using relevant MeSH terms. Details on our search strategy can be found in Online Resource 1. In addition, the reference lists of the (review) articles identified through MEDLINE and PsycINFO were reviewed for additional studies. In addition to the studies identified through systematic review, to make the publication list as comprehensive as possible, studies were also added by the authors (i.e. through precedent knowledge about relevant publications). Shortly prior to publication, we updated the literature search to also include studies between June 2019 and May 2021. The methodology of the ESSTS survey is presented in an editorial in the current issue of this journal.

## Results

### Evidence-based psychological interventions

In the 2011 European clinical guidelines, behavior therapy (BT) was recommended as a first-line intervention for tic disorders in children and adults [5, 6]. The rationale for using BT for treating tic disorders is based on the fact that tics can be suppressed for various lengths of time, and that the expression of tics, beyond their neurobiological origin, is influenced by contextual factors. These contextual factors include the perception of premonitory urges and other internal (e.g. emotional) states and environmental contingencies (e.g. specific situations or activities, stress-inducers, social reactions). The goal of BT is to provide patients with tic-specific behavioral techniques to enhance self-control and to decrease factors that worsen or maintain tics.

Of the different BT interventions available, most experimental evidence was found for *Comprehensive Behavioral Intervention for Tics* (CBIT), where *Habit Reversal Training* (HRT) is considered the main component [7, 8]. Evidence was also found for *Exposure and Response Prevention* (ERP) [9]. The 2011 European clinical guidelines recommended both HRT/CBIT and ERP as first-line interventions. In 2012, Canadian clinical guidelines were published, which also recommended BT as a first-line intervention for tic disorders, stating that especially CBIT is supported by strong evidence for efficacy and safety [10]. Recently, the American Academy of Neurology (AAN) published their clinical guidelines for the treatment of tic disorders, again recommending BT as a first-line intervention for tic disorders. The AAN guidelines specify that clinicians should offer CBIT as an initial treatment option prior to other psychological interventions and to pharmacotherapy (PT). If CBIT is unavailable, ERP may be an acceptable alternative. According to the AAN guidelines, CBIT is the only intervention to achieve the highest rating (“high confidence” to reduce tics compared to the control condition), which was not achieved by any of the PTs [11].

The following sections describe clinical trials of psychological interventions, published since the 2011 European clinical guidelines, based on our current literature search. Table 1 presents details on randomized controlled trials (RCTs) published since 2011, as well as influential RCTs published prior to 2011. Treatments for tic disorders that were mentioned in the 2011 European clinical guidelines, but were not examined or supported by RCTs (such as *massed negative practice*, *self-monitoring and relaxation training*), and where no new substantial evidence has been published since are not covered in this update [5].

**Table 1:** Randomized controlled trials of psychological interventions for tic disorders published since 2011, as well as a selection* of previous influential trials to enable comparison

Study	Groups	Subjects	Efficacy	Follow-up	Comments
Verdellen et al. (2004) [9]	HRT vs ERP	*N* = 43 Age 7–55	**YGTSS-TTS**: HRT: 24.1 (pre) to 19.7 (post) Within-group effect size: 1.06 ERP: 26.2 (pre) to 17.6 (post) Within-group effect size: 1.42	**3FU**: *YGTSS-TTS*: HRT: 13.5 ERP: 14.0	**Duration**: HRT: 10 sessions (1 h, weekly); ERP: 12 sessions (2 h, weekly) **Results**: No significant between-group effect on the YGTSS-TTS. Significant within-group effects on the YGTSS-TTS for each group **Follow-up**: Treatment effect maintained for both groups **Limitations**: Uncertain whether the study was powered to detect significant between-group effects. 3FU included 25 participants who had been crossed over to the other group
Piacentini et al. (2010) [8]	CBIT vs PST	*N* = 126 Age 9–17	**YGTSS-TTS**: CBIT: 24.7 (pre) to 17.1 (post) PST: 24.6 (pre) to 21.1 (post) Between-group effect size: 0.68	**6FU**: CBIT: 20 out of 23 available initial treatment responders were still classified as treatment responders (according to the CGI-I)	**Duration**: 8 sessions (during 10 weeks; first two sessions 1.5 h, remaining sessions 1 h). 3 monthly booster sessions for responders **Interventions**: CBIT included psychoeducation about tic disorders, HRT, relaxation training, and functional analysis; PST included psychoeducation and supportive psychotherapy **Results**: Significant between-group effect on the YGTSS-TTS (in favor of CBIT) **Follow-up**: Treatment effects were maintained **Limitations**: Follow-up data were only available for initial treatment responders
Himle et al. (2012) [54]	F2F CBIT vs VC CBIT	*N* = 20 Age 8–17	**YGTSS-TTS**: F2F CBIT: 24.1 (pre) to 17.6 (post) VC CBIT: 23.4 (pre) to 15.6 (post)	**4FU**: *YGTSS-TTS:* F2F CBIT: 20.1 VC CBIT: 16.8	**Duration**: 8 sessions (weekly) via a VC software **Interventions**: CBIT included psychoeducation about tic disorders, HRT, relaxation training, and functional analysis **Results**: No significant between-group effect on the YGTSS-TTS. Significant within-group effects on the YGTSS-TTS for each group **Follow-up**: Significant within-group effect from pre to 4FU for the combined sample **Limitations**: Uncertain whether the study was powered to detect significant between-group effects
Wilhelm et al. (2012) [15]	CBIT vs PST	*N* = 122 Age 16–69	**YGTSS-TTS**: CBIT: 24.0 (pre) to 17.8 (post) PST: 21.8 (pre) to 19.3 (post) Between-group effect size: 0.57	**6FU**: CBIT: 12 out of 15 available initial treatment responders were still classified as treatment responders (according to the CGI-I)	**Duration**: 8 sessions (during 10 weeks; first two sessions 1.5 h, remaining sessions 1 h). 3 monthly booster sessions for responders **Interventions**: CBIT included psychoeducation about tic disorders, HRT, relaxation training, and functional analysis; PST included psychoeducation and supportive psychotherapy **Results**: Significant between-group effect on the YGTSS-TTS (in favor of CBIT). Significantly higher proportion of treatment responders in the CBIT group (24 vs. 4), as measured by the CGI-I **Follow-up**: Treatment effects were maintained **Limitations**: Follow-up data were only available for initial treatment responders
McGuire et al. (2015) [22]	LWT vs Waitlist	*N* = 24 Pediatric sample	**YGTSS-TTS**: LWT: 20.2 (pre) to 14.3 (post) Waitlist: 24.7 (pre) to 24.8 (post) **YGTSS Impairment Score**: LWT: 27.5 (pre) to 8.3 (post) Waitlist: 31.7 (pre) to 23.8 (post) Between-group effect size: 1.50	**1FU**: 1FU was completed for 5 out of 10 initial treatment responders (according to the CGI-I). No change was found between the post and 1FU YGTSS Impairment scores, indicating maintenance of the treatment effects	**Duration**: 10 sessions (weekly) **Interventions**: LWT included abbreviated HRT, cognitive restructuring, problem solving, parent training, emotion regulation, overcoming tic-related avoidance, talking about tics with peers and coping at school, and improving self-esteem **Results**: Significant between-group effect on the YGTSS Impairment score (primary outcome) (in favor of LWT). No significant between-group effect on the YGTSS-TTS, but significant within-group effect for the LWT group on the same measure **Follow-up**: Treatment effects were maintained **Limitations**: Follow-up data were only available for initial treatment responders
Ricketts et al. (2016) [55]	VC CBIT vs Waitlist	*N* = 20 Age 8–16	**YGTSS-TTS**: VC CBIT: 25.8 (pre) to 18.5 (post) Waitlist: 22.0 (pre) to 20.3 (post) Between-group effect size: 0.15 (partial η^2^)	**FU**: N/a	**Duration**: 8 sessions (during 10 weeks; first two sessions 1.5 h, remaining sessions 1 h) via a VC software **Interventions**: CBIT included psychoeducation about tic disorders, HRT, relaxation training, and functional analysis **Results**: Significant between-group effect on the YGTSS-TTS (in favor of VC CBIT)
Yates et al. (2016) [49]; Dabrowski et al. (2018) [50]	Group HRT vs Group PE	*N* = 33 Age 9–13	**YGTSS-TTS**: N/a **YGTSS-MTSS**: Group HRT: 17.7 (pre) to 15.1 (post) Group PE: 16.3 (pre) to 15.9 (post) Between-group effect size: 0.55	**12FU**: *YGTSS-MTSS:* Group HRT: 12.2 Group PE: 13.8	**Duration**: 8 sessions (first two sessions 1.5 h, remaining sessions 1 h) delivered in a group format **Results**: Significant between-group effect on the YGTSS-MTSS (in favor of Group HRT) **Follow-up**: Both groups combined improved significantly on the YGTSS-TTS and the YGTSS-MTSS between pre and 12FU **Limitations**: YGTSS-TTS were not reported at pre and post
Seragni et al. (2018) [19]	HRT vs UC	*N* = 21 Pediatric sample	**YGTSS-TTS**: N/a	**3FU**: *YGTSS-MTSS:* Significant within-group effect for HRT and UC combined into one group. Scores n/a	**Duration**: HRT: 8 sessions (during 10 weeks); UC: 3 sessions (during 10 weeks) **Results**: No significant between-group effect on any reported YGTSS score **Follow-up**: Significant within-group effect for HRT and UC combined into one group **Limitations**: High number of dropouts, only 13 participants completed the trial. Uncertain whether the study was powered to detect significant between-group effects. Effect sizes were not reported
Rizzo et al. (2018) [14]	BT vs. PE PT vs. PE BT vs. PT	*N* = 110 Age 8–17	**YGTSS-TTS**: BT: 19.8 (pre) to 11.4 (post) PT: 24.1 (pre) to 15.7 (post) PE: 22.0 (pre) to 21.7 (post)	**FU**: *YGTSS-TTS at 3 months post BT/PE and 5 months post initiation of PT:* BT: 12.4 PT: 14.7 PE: 20.7	**Duration**: 8 sessions (weekly, first two sessions 1.5 h, remaining sessions 1 h) **Interventions**: BT included HRT or ERP; PT included risperidone, aripiprazole or pimozide **Results**: Significant between-group effects on the YGTSS-TTS for BT vs. PE and PT vs. PE (in favor of BT and PT) **Limitations**: Uncertain whether the study was powered to detect significant between-group effects between BT and PT. Effect sizes were not reported
Andrén et al. (2019) [59]	Internet ERP vs. Internet HRT	*N* = 23 Age 8–16	**YGTSS-TTS**: Internet ERP: 23.8 (pre) to 18.3 (3FU) Within-group effect size: 1.12 Internet HRT: 23.5 (pre) to 20.2 (3FU) Within-group effect size: 0.50	**12FU**: *YGTSS-TTS:* Internet ERP: 16.9 Internet HRT: 19.4	**Duration**: 10 weeks of therapist-supported (via text messages) internet-delivered treatment **Interventions**: In addition to the ERP or HRT core elements, both groups included functional analysis and parent training **Design**: Study did not aim to compare groups **Results**: Significant within-group effect on the YGTSS-TTS for the Internet ERP group, but not the Internet HRT group **Follow-up**: Effects were maintained at 12FU
Nissen et al. (2019, 2021) [43, 52]	Individual HRT + ERP vs Group HRT + ERP	*N* = 59 Age 9–17	**YGTSS-TTS**: Individual HRT + ERP: 23.8 (pre) to 14.3 (post) Within-group effect size: 1.21 Group HRT + ERP: 23.4 (pre) to 15.9 (post) Within-group effect size: 1.38	**12FU**: *YGTSS-TTS:* Individual HRT + ERP: 12.7 Group HRT + ERP: 12.8	**Duration**: 8 regular sessions and 1 booster session, delivered individually or in a group format **Results**: No significant between-group effect on the YGTSS-TTS. Significant within-group effects on the YGTSS-TTS for each group **Follow-up**: Treatment effects were maintained for both groups at 12FU **Limitations**: Uncertain whether the study was powered to detect significant between-group effects
Chen et al. (2020) [63]	CBIT + UC vs. UC	*N* = 46 Age 6–18	**YGTSS-TTS**: CBIT: 19.3 (pre) to 10.4 (post) UC: 17.7 (pre) to 14.5 (post) Between-group effect size: 0.56	**3FU**: *YGTSS-TTS:* CBIT: 6.6	**Duration**: CBIT: 4 sessions (during 3 months) **Interventions**: CBIT included psychoeducation, habit reversal training, relaxation training, and relapse prevention; UC included psychoeducation and 50 mg of pyridoxine (per day) **Results**: Significant between-group effect on the YGTSS-TTS (in favor of CBIT + UC) **Follow-up**: Further improvement for the CBIT + UC-group in a within-group analysis at 3FU **Limitations**: No intention-to-treat analysis
McGuire et al. (2020) [33]	HRT + DCS vs. HRT + placebo	*N* = 20 Age 8–17	**YGTSS-TTS**: N/a	**FU**: N/a	**Duration**: HRT: 1 session **Interventions**: HRT; 50 mg of DCS or placebo. DCS was hypothesized to enhance the effect of HRT **Results**: Significant between-group effect on the Hopkins Motor/Vocal Tic Scale (in favor of HRT + DCS), for the two bothersome tics targeted in treatment **Limitations**: Did not use the YGTSS. Low dose of HRT compared to previous trials (only 1 session)
Rachamim et al. (2020) [60]	Internet CBIT vs. Waitlist	*N* = 41 Age 7–18	**YGTSS-TTS**: Internet CBIT: 22.7 (pre) to 16.1 (post) Waitlist: 21.9 (pre) to 20.9 (post) Between-group effect size: 0.20 (partial η^2^)	**6FU**: *YGTSS-TTS:* Internet CBIT: 11.0	**Duration**: 9 weeks of therapist-supported (via telephone) internet-delivered treatment + 6 monthly booster sessions **Results**: Significant between-group effect on the YGTSS-TTS (in favor of Internet CBIT) **Follow-up**: Large within-group effect for the Internet CBIT group at 6FU
Singer et al. (2020) [56]	DVD HRT vs. HRT	*N* = 44 Age 7–13	**YGTSS-TTS**: DVD HRT: 27.8 (pre) to 18.8 (post) HRT: 28.2 (pre) to 20.7 (post)	**FU**: N/a	**Duration**: HRT: 8 sessions (during 10 weeks) **Interventions**: DVD HRT-group received a DVD and written instructions on how to use HRT at home with the support of a parent; HRT-group received regular face-to-face HRT **Results**: No significant between-group effect. Significant within-group effects in both groups **Limitations**: Uncertain whether the study was powered to detect significant between-group effects. Large dropout rates and the lack of an intention-to-treat analysis make results difficult to interpret
Zimmerman-Brenner et al. (2021) [51]	Group CBIT vs. Group PE	*N* = 61 Age 8–15	**YGTSS-TTS**: Group CBIT: 24.8 (pre) to 39.8 (post) Group PE: 22.0 (pre) to 37.1 (post)	**3FU**: *YGTSS-TTS:* Group CBIT: 18.4 Group PE: 21.8	**Duration**: 8 weekly sessions (first two sessions 1.5 h, remaining sessions 1 h; considered the acute treatment phase) + 3 monthly 1-h booster sessions (during the follow-up phase). All sessions were delivered in a group format **Results**: No significant between-group effect. Significantly increased YGTSS-TTS in both groups (within-group analysis) at post-treatment, seemingly driven by increased vocal tic severity **Follow-up**: Significantly decreased YGTSS-TTS in both groups (within-group analysis) when comparing baseline to the 3-month follow-up **Limitations**: Uncertain whether the study was powered to detect significant between-group effects. No intention-to-treat analysis

*The previous trials were selected following expert consensus as especially important for the recommendations of the initial European clinical guidelines published in 2011

*1–3−4–6−12FU*  1–3-4–6-12 months post-treatment time-point follow-up; *BT *  behavior therapy; *CBIT*  comprehensive behavioral intervention for tics; *CGI−I* Clinical Global Impressions—Improvement scale; *CTD* chronic tic disorder; *DCS* d-cycloserine; *ERP* exposure and response prevention; *F2F* face-to-face; *FU*  follow-up; *HRT*  habit reversal training; *LWT* Living with tics; *N/a *  not available; *PE*  psychoeducation; *post *  post-treatment time-point; *pre*  pre-treatment/baseline time-point; *PST* psychoeducation and supportive psychotherapy; *PT* pharmacotherapy; *TS* Tourette syndrome; *UC*  usual care; *VC*  videoconference; *YGTSS*  Yale Global Tic Severity Scale; *YGTSS−MTSS* Yale Global Tic Severity Scale—Motor Tic Severity Score; *YGTSS−TTS*  Yale Global Tic Severity Scale—Total Tic Severity Score

### Psychoeducation

Psychoeducation refers to the clear sharing of understandable, up-to-date information about the symptoms, cause, prognosis, potential management, treatment and daily experience of a condition. Such information is typically included as a first step in various treatment protocols of evidence-based psychological interventions for tic disorders (e.g. [12, 13]). However, as a stand-alone intervention aimed at reducing tic severity, psychoeducation (sometimes also extended with information on healthy habits and common comorbid conditions, and referred to as *psychoeducation and supportive psychotherapy* [PST]) has been shown inferior to BT and PT in several RCTs [8, 14, 15]. In a review of psychoeducation for teachers and peers, it was concluded that psychoeducation increases knowledge, positive attitudes and behaviors towards individuals with TS [16].

Despite psychoeducation being described in clinical guidelines as a first important step of any treatment for TS [5, 11], evidence on what specific elements should be addressed is lacking. A comprehensive overview of suggested information to include can be found in a review by Wu and McGuire [17].

### Habit reversal training (HRT) and Comprehensive behavioral intervention for tics (CBIT)

HRT consists of two primary parts: First, awareness training, which includes different techniques to increase awareness of tic expression and associated premonitory urges. Second, competing response training, in which physically incompatible responses are identified and applied, which prevent tics from being expressed. In HRT, tics are treated on a one-by-one basis. All current tics are listed and rated in terms of their severity. Typically, the most bothersome tic from this hierarchy is selected to be treated first. This tic is then subjected to awareness training, in which the patient learns to detect when the tic is occurring, as well as the signals that precede tic. Once a patient has developed a good awareness of the tic and can predict the occurrence of the tic, competing response training begins. Competing response training involves the selection and subsequent implementation of a physically incompatible behavior designed to prevent tics from occurring. The competing response generally employs the same muscles as the tic and should be able to be performed for a sustained period. Once a competing response has been practiced in a session, the patient continues to practice it at home. As soon as the patient learns to use the competing response to reliably prevent the tic, the treatment focus is shifted to the next tic in the hierarchy [12, 18]. CBIT is an expanded version of HRT, and additionally includes therapeutic strategies such as relaxation training, contingency management, and interventions based on functional analyses to address contextual factors which influence tic expression [8, 12].

The 2011 European clinical guidelines reported several RCTs of HRT/CBIT, demonstrating medium to large treatment effects. The largest RCT evaluated CBIT in 126 children (9–17 years) with TS or CTD [8]. In this study, CBIT was superior to psychoeducation and PST in reducing tic severity (as measured by the Yale Global Tic Severity Scale - Total Tic Severity Score [YGTSS-TTS]; effect size: 0.68, as compared to PST). In a 6-month follow-up of treatment responders (defined as a score <3 on the Clinical Global Impressions–Improvement Scale [CGI-I]) of both groups, treatment gains were shown to be maintained for the majority of the responders in a completer analysis. In parallel to this trial, Wilhelm et al. [15] published an RCT in 2012 comparing CBIT with PST in 122 adults (16–69 years) with TS or CTD. In line with the pediatric trial, all patients received eight sessions of either condition, while responders additionally received three monthly booster sessions. As in the pediatric trial [8], CBIT was found to be superior to PST (effect size: 0.57). The responder rate (defined as CGI-I <3) was, however, lower in the adult trial (38.1% compared to 52.5% in the pediatric trial), which was hypothesized to reflect that the adult participants suffered from a more treatment-resistant form of the disorder. The overall dropout rate was 13.9%, with no difference between groups. Treatment responders of both groups continued to show benefits up to the 6-month follow-up, in a completer analysis.

The literature search also identified a few smaller clinical trials of HRT. Seragni et al. conducted a randomized pilot study (*N*=21) comparing HRT with a control condition (three sessions of routine treatment with a neuropsychiatrist, without prescription of PT) for young people with TS [19]. Participants showed an improvement in tic reduction and global functioning in both groups, without significant between-group differences. Interpretation of the results was hampered by the small sample size and a high number of dropouts in both groups. Viefhaus et al. examined the efficacy of a German BT program (similar to CBIT) including HRT, psychoeducation and additional behavioral interventions (e.g. functional interventions) for young people (8–16 years; *N*=27) with TS/CTD [20]. In a within-group design (8 weeks pre-treatment; 16 sessions treatment), significant improvements were found on tic severity (YGTSS-TTS, within-group effect size: 0.89) and tic-related impairment (YGTSS Impairment Score, within-group effect size: 0.31) at post-treatment. Bennett et al. evaluated a modified version of CBIT for use among very young patients (5–8 years) in an open study [21]. Compared to the previously published CBIT protocol [12], the adaptations included fewer sessions (six instead of eight), larger parent involvement, and a simplified explanation of HRT through playing cards picturing body movements and competing responses. The results showed a medium-sized, significant within-group effect (*d*=0.73) on the YGTSS-TTS at post-treatment, which later was maintained at a 12-month follow-up. The study provides preliminary evidence for CBIT also being efficacious in this younger patient group.

Further adaptations to BT have been made to broaden the focus from reducing tic severity to improving the individual’s overall quality of life. McGuire et al. evaluated a modular treatment protocol (“Living with Tics”; LWT) that incorporates HRT with psychoeducation, problem-solving, distress tolerance, and coping at school, with the aim of improving resilience and reducing tic-related impairment [22]. Preliminary findings of this intervention in youth (*N*=24) showed the LWT intervention to be efficacious in improving quality of life relative to a waitlist control (YGTSS Impairment Score, effect size: 1.50). Ten participants (83%) in the LWT condition were rated as treatment responders, compared to four participants (33%) in the waitlist condition. Treatment gains were maintained at a 1-month follow-up [22].

To summarize, several RCTs support the use of HRT/CBIT as an effective treatment for tics in children and adults with TS.

### Exposure and response prevention (ERP)

Similar to HRT, ERP is based on learning theory. In ERP, the individual practices suppressing tics for prolonged periods of time (response prevention), with gradually increased exposure to premonitory urges and environmental factors (e.g. situations and activities) that are likely to induce tics, with the aim to increase urge tolerance and thereby reduce tics. Unlike HRT, no tic hierarchy needs to be created and all tics are worked with at the same time. In ERP, the patient is first trained to enhance tic suppression. A stopwatch is used to record tic suppression times and the patient is motivated to beat his/her record on each new trial. In the next phase, exposure is optimized by focussing on the premonitory urges, being exposed to stimuli that are known to elicit tics and practicing in various situations and activities. Meanwhile, the patient is instructed to keep resisting all tics. Apart from the in-session training, the patient is encouranged to continue practicing ERP on his/her own between the sessions [9, 23].

In the 2011 European clinical guidelines part III on behavioral interventions one RCT of ERP for the treatment of tic disorders was reported [9], where 43 children and adults (7–55 years) were randomized to either ERP or HRT. The results demonstrated comparable effects for both treatments (within-group effect sizes: 1.42 for ERP and 1.06 for HRT). Results were maintained up to a 3-month follow-up, but the interpretation is hampered by cross over between treatments. Since 2011, only open studies have been published examining the treatment effects of ERP. In a naturalistic study by Andrén et al. [24], 74 participants (6–17 years) received BT at a TS specialist clinic in Sweden. Out of the 74 participants, 46 received ERP, 14 received HRT, and 14 received various combinations of the two. Results showed a significant and large within-group effect (*d*=1.03) on the YGTSS-TTS for the combined BT group at post-treatment, with further improvement at a 12-month follow-up. The study provides some additional open data on the efficacy of mainly ERP, but primarily the authors conclude that BT can be delivered in a naturalistic specialist clinical setting, with comparable effects to RCTs.

### Cognitive interventions

To date, there are no RCT data supporting cognitive interventions as a stand-alone treatment for TS. Since the 2011 European clinical guidelines, a new treatment model has been proposed by O'Connor et al. involving cognitive-behavioral and psychophysiological elements [25]. This model describes an association between maladaptive beliefs about tics, premonitory urges, perfectionistic personality traits and negative psychophysiological consequences, such as elevated muscle tension in body areas where tics occur. The cognitive psychophysiological treatment developed by O’Connor et al. is a combination of sensorimotor activation and (meta-) cognitive interventions to target the proposed affected areas. So far, two open trials have been published in 36 adults and seven children with TS, indicating tic severity reduction after treatment [25, 26]. While being a possibly promising new treatment approach, RCT data are needed to determine the treatment effects.

### Third-wave interventions

Third-wave interventions represent both an extension of and deviation from traditional cognitive-behavioral approaches, and include concepts such as metacognitive training, mindfulness and psychological flexibility, as part of behavioral treatments. The acceptance-based approach, which is shared by several third-wave interventions, prioritizes the promotion of health and well-being and suggests that rather than trying to control aversive psychological, emotional or physiological symptoms, accepting them might reduce their negative impact. [27]. So far, only a few studies have targeted the feasibility and efficacy of third-wave interventions for the treatment of patients with TS. A pilot study by Franklin et al. evaluated the feasibility of a combined treatment of HRT and acceptance and commitment therapy (ACT) in a small sample of adolescents with TS/CTD (*N* = 13; 14–18 years), showing comparable results to traditional HRT [28]. Reese et al. tested the feasibility and efficacy of a modified form of mindfulness-based stress reduction (MBSR-tics) in a small open trial of adolescents and adults (16–67 years; *N* = 18) with TS/CTD [29]. Fifty-nine percent of the participants were classified as treatment responders and results were maintained up to the 1-month follow-up. In a later study, Reese et al. modified the MBSR-tics intervention for online delivery [30]. In this open study (26–59 years; *N* = 5), the intervention was judged feasible and acceptable. However, effects on tic severity and tic-related impairment from baseline to post-treatment were modest. The authors especially point out that participant adherence to homework assignments, in this online format, was lower than anticipated.

The acceptance-based approach has also been tested with a focus on premonitory urge sensations. In an experimental study, 45 young people (8–17 years) participated in three different two-minute-conditions: free-to-tic (baseline), tic suppression and urge acceptance [31]. Results showed a significantly higher decrease in frequency and intensity of premonitory urges in the urge acceptance condition, compared to the other conditions. Additionally, the level of discomfort was found to be significantly lower during the urge acceptance condition compared to the tic suppression condition.

Another third wave intervention is *resource activation*, which has been evaluated in a within-subject pilot trial for young people (8–19 years; *N* = 24) with TS/CTD [32]. The treatment focuses on the strengths and abilities of the patients and includes relaxation and mindfulness techniques. The trial showed significant reductions of tic severity and tic-related impairment, indicating that resource activation is a potentially effective treatment for patients with TS.

These pioneer studies indicate the potential feasibility of third-wave interventions for TS, however, RCTs are needed to determine efficacy and make recommendations for their use. 

### BT and PT

Only one RCT comparing the effects of BT to PT on tic severity has been published to date. Rizzo et al. randomized 110 young people (8–17 years) into three groups: BT (either HRT or ERP), PT (either risperidone, aripiprazole or pimozide), and psychoeducation [14]. Data were available for 102 participants (BT: *n*=25; PT: *n*=53; psychoeducation: *n*=24). At post-treatment, tic severity (as measured by the YGTSS-TTS) improved significantly in the BT and PT groups compared to the psychoeducation group (between-group effect sizes: 1.42 for BT and 0.84 for PT [calculated from data presented in the original article]). The larger effect size in the BT group compared to the PT group may partially be explained by differences in baseline tic severity. In the same vein, there were no significant differences between the BT and PT groups at the same measure, indicating that BT and PT potentially could be equally effective. While these results are important, replication studies are warranted given the limitations of this RCT. These include low statistical power to assess between-group differences in the three conditions and the lack of intention-to-treat data.

Originating from animal study findings, cognitive enhancers such as D-cycloserine (DCS) are hypothesized to strengthen newly learned associations, which in turn may augment the treatment effects of BT. In a preliminary RCT [33], McGuire et al. randomized 20 participants (8–17 years) to one session of HRT plus 50 mg of DCS or one session of HRT plus placebo. The study found a significant between-group effect (in favor of the HRT plus DCS-group) on the Hopkins Motor/Vocal Tic Scale, for the two bothersome tics targeted in the HRT treatment. Limitations include not providing a full dose of HRT treatment and not including the YGTSS as an outcome measure. Further studies are needed to establish the possibly augmenting effect of DCS on BT.

### Meta-analyses of BT

In recent years, as more RCTs on the efficacy of BT have been published, a number of systematic reviews and meta-analyses have been undertaken [34–38]. The studies range from an early meta-analysis by Wile et al. [34] including 4 RCTs to the most recent meta-analysis by Yu et al. [38], which summarized 10 RCTs exclusively of HRT and CBIT. The latter meta-analysis included a total of 586 participants and found a medium effect size for HRT (SMD = 0.43). Additional subgroup analyses indicated no differences in the therapeutic effect comparing mode of delivery (face-to-face vs. online) or age group (children vs. adults). Notably, Yu et al. defined strict inclusion criteria, such as only including studies which employed the YGTSS, thus resulting in some earlier trials being excluded (e.g. [39]). A meta-analysis by McGuire et al. [35], which was published 6 years earlier, employed less strict criteria (summarizing 8 RCTs, with *N* = 438), and reported a slightly larger medium effect size (SMD = 0.67) for BT.

Regarding other types of psychological interventions, a meta-analysis by Hollis et al. [36] found no evidence for tic-specific effectiveness of relaxation training, parent training, or anger control training.

### Predictors and moderators of response to BT

A few studies have examined, primarily in a post-hoc fashion, predictors and moderators of response to BT for tic disorders. In a meta-analysis including pediatric and adult trials, McGuire et al. found that BT had larger treatment effects among trials with older average participant age, more therapy sessions, and with less co-occurring attention-deficit/hyperactivity disorder (ADHD), while concurrent PT for TS did not influence the treatment effects [35]. However, findings regarding the impact of ADHD on therapy are equivocal. Conelea et al. [40], using data from experimental settings, showed that young people (5–17 years) with ADHD can suppress tics just as effectively as those without ADHD.

Sukhodolsky et al. examined predictors and moderators of treatment in BT and PST [41]. The study showed that positive participant expectancy and greater tic severity predicted greater tic improvement in both groups, while comorbid anxiety disorders and greater premonitory urge severity predicted a lower tic improvement [41]. The presence of PT for TS predicted tic reduction in the PST group, but not in the BT group. Taken together, the available studies suggest that PT for TS does not influence the treatment effects of BT. In another study, based on data from the same original RCTs as used in the Sukhodolsky et al. study, Essoe et al. concluded that adherence to homework assignments predicted tic reductions and treatment response [42].

Using data from a randomized trial evaluating a combination of HRT and ERP (described in more detail in a later section) [43], Nissen et al. investigated possible predictors and moderators of treatment response [44]. Their data suggest that internalizing symptoms (anxiety) predicted a lesser reduction in functional impairment and that participants’ (negative) beliefs about their tics were shown to have a negative effect on treatment outcome.

More studies are needed to replicate and further deepen the understanding of potential predictors and moderators of response to BT for patients with TS.

### Neurobiology of BT

So far, only one study has investigated neurobiological changes following the use of BT in TS. Deckersbach et al. [45] used functional magnetic resonance imaging (fMRI) to investigate 8 subjects who participated in a large CBIT trial [15] matched with 8 healthy controls. fMRI was conducted pre- and post-treatment in conjunction with a visuospatial priming task to measure response inhibition. The authors found a decrease of striatal activation in the putamen at the post-treatment assessment, which formed a hypothesis that BT leads to a normalization of activation in the putamen. A further finding was a negative correlation between change in tic severity (as measured by the YGTSS-TTS) and a region in the inferior frontal gyrus. A similar more recent study by Petruo et al. [46] used an inhibitory control task to investigate the hypothesis that patients with TS (*n* = 21) exhibit an increased perception–action binding [47] as compared to healthy controls (*n* = 21). Indeed, patients exhibited an impaired performance on the task at baseline, which was normalized after the CBIT intervention.

### Novel modalities of established behavioral treatments

Given the limited availability of therapists trained in delivering BT for patients with tic disorders [48], focus on dissemination and adaptation of treatment delivery has increased in recent years. New modalities have been proposed to make BT more accessible, primarily by reducing the number of therapists needed and/or reducing the need for travel. The modalities fall into three main areas: group delivery; videoconference delivery; and internet delivery. Additionally, there are case series using intensive treatment delivery schedules to reduce travel time.

### Group delivery of BT

Group delivered BT for patients with TS has emerging evidence to date. Yates et al. compared two 8-session group interventions (CBIT [*n* = 17] vs psychoeducation [*n* = 16]) among children (9–13 years) with TS [49]. The results showed a reduction in motor tic severity at post-treatment (effect size: 0.55, in favor of the CBIT group). None of the groups showed a significant reduction in vocal tic severity. The observed treatment effects on tic severity and quality of life were maintained at a 12-month follow-up [50]. Interestingly, both groups reported a higher rate of school attendance in the year following treatment as compared with the year before the intervention. In this study, meeting other young people with tics did not increase tic expression, which is a common fear expressed by parents and patients.

Zimmerman-Brenner et al. [51] compared group-delivered CBIT to group-delivered psychoeducation in a RCT (8–15 years; *N* = 61). Participants received 8 weekly sessions during the acute treatment phase and 3 additional monthly sessions during a 3 months follow-up phase. Results showed no significant between-group effect on the YGTSS-TTS at post-treatment, but significant within-group improvements on the YGTSS Motor Tic Severity Score and the YGTSS Impairment Score for both groups. Interestingly, tic severity as measured by the YGTSS-TTS increased in both groups at post-treatment. This effect was seemingly driven by a significant increase in vocal tic severity, which could have been a side effect of the group format. At the 3-month follow-up, however, both groups showed improved YGTSS-TTS scores compared to baseline, indicating that the worsened vocal tic severity was temporary. Further, only the CBIT group showed a maintained improvement on the YGTSS Motor Tic Severity Score at the 3-month follow-up, indicating a possible benefit for this active treatment.

Nissen et al. conducted a randomized trial comparing a combination of HRT and ERP in young people (9–17 years; *N* = 59) in either an individual setting or a group setting [43]. Both settings involved nine sessions, where HRT was introduced before ERP, and the final sessions were devoted to the type of BT that seemed most effective for that specific participant. The study showed significant tic severity reductions in both settings (within-group effect sizes: 1.21 for the individual setting and 1.38 for the group setting). A total of 66.7% of the participants were considered treatment responders (defined as a 25% reduction on the YGTSS-TTS). There was no statistically significant difference between the groups, apart from the YGTSS Impairment Score (in favor of the individual setting). The within-group treatment effects were maintained for both groups at a 12-month follow-up [52].

Lastly, in an open pilot study by Heijerman-Holtgrefe et al. [53] (9–14 years; *N* = 14), ERP was evaluated in an intensive group format (12 sessions fitted into 3 + 1 days). This so-called *“Tackle your tics”*-programme further included coping strategy workshops led by young adult patients, relaxation training, and separate parent meetings. The results showed a significantly decreased tic severity (YGTSS-TTS) between baseline and a 2-month follow-up (*η*_p_^2^ = 0.41), increased quality of life and high treatment satisfaction.

To summarize, studies of group delivery of BT have shown mixed results. More studies are needed to make firm recommendations for clinical practice.

### Videoconference delivery of BT

Videoconference BT is identical to regular face-to-face BT, except for that the (real time) communication between the patient and therapist is made via videoconference software. Two pilot RCTs have evaluated CBIT via videoconference delivery [54, 55]. Himle et al. compared videoconferencing (received at a clinic) to face-to-face delivery (8–17 years; *N* = 20) and found that tic severity was reduced regardless of the CBIT modality, with similar within-group effects at a 4-month follow-up [54]. Ricketts et al. compared videoconferencing (received at home via the software Skype) to a waiting-list control condition (8–16 years; *N* = 20), and found a greater tic severity reduction in the videoconferencing group, compared to the waiting list condition [55]. Although some challenges (like video/audio problems and difficulties viewing homework) were described [55], both studies reported strong therapeutic alliance ratings, treatment satisfaction, and videoconferencing satisfaction in the videoconferencing groups [54, 55]. These findings suggest that videoconferencing is a feasible and acceptable format for the delivery of BT for young people with TS. Larger controlled studies are, however, needed to determine the clinical efficacy of this format.

A perhaps related treatment delivery format, where a DVD is provided to the patient with instructions on how to perform HRT (with support of a parent), has been tested in a pilot randomized controlled trial (7–13 years; *N* = 44) [56]. Both the DVD-HRT group and the comparison face-to-face-HRT group showed improvements on the YGTSS-TTS in a within-group analysis. Results are, however, difficult to interpret due to large dropout rates and the lack of an intention-to-treat analysis.

### Internet delivery of BT

In internet-delivered BT, patients work through a self-help programme briefly supported by a therapist (via text messages or telephone). A Swedish internet platform called BIP (Barninternetprojektet [The Child Internet Project]) has successfully been used to deliver such internet-delivered treatment for several pediatric mental health conditions [57, 58]. Andrén et al. used the BIP-platform to evaluate two therapist-guided internet-delivered interventions based on HRT and ERP principles (called BIP TIC HRT and BIP TIC ERP) in a pilot trial (8–16 years; *N* = 23) [59]. Both interventions showed a significant reduction in tic-related impairment and parent-rated tic severity, but only BIP TIC ERP showed a significant improvement in clinician-rated tic severity as assessed by the YGTSS-TTS (within-group effect sizes at the 3-month follow-up: BIP TIC ERP: 1.12; BIP TIC HRT: 0.50). Therapeutic gains were maintained at the 12-month follow-up. An additional advantage of the treatment format was that it demanded less therapist time (approximately an average of 25 min per participant per week, mainly via text messages) than traditional face-to-face BT.

In an Israeli RCT (7–18 years; *N* = 45) [60], Rachamim et al. compared internet-delivered CBIT to a waitlist condition. The results showed a large, significant between-group effect on the YGTSS-TTS (*η*_p_^2^ = 0.20; in favor of internet-delivered CBIT). The active group was followed until 6 months post-treatment, where it showed a large within-group effect on the YGTSS-TTS (*d* = 2.25). Also in this study, therapists spent considerably less time with patients (ca. 7 min per participant per week, via telephone) than in traditional face-to-face BT.

### Treatment intensity

Studies have explored the benefits of delivering treatment in an intensive and brief manner, potentially making treatment more efficient and convenient for patients who travel long distances to receive care. Blount et al. piloted an intensified version of the CBIT-protocol (several hours of daily treatment over a 4 day period, called IOP CBIT) in two boys (ages 10 and 14 years) with TS, showing a tic reduction which was maintained up to 6 and 7 months later [61]. Along the same line, van de Griendt et al. addressed the question of whether shorter sessions of ERP (1 h compared to the 2 h used in the Verdellen et al. RCT [9]) would yield a different treatment outcome [62]. Results suggest that shorter sessions were not inferior to longer sessions regarding tic severity outcomes, implicating the clinical use of shorter sessions to accommodate more treatment delivery within the same time frame [62]. Chen et al. evaluated the effects of a shortened CBIT-protocol (four instead of eight sessions). In a RCT [63], 46 participants (6–18 years) were randomized to shortened CBIT plus usual care (psychoeducation and 50 mg of pyridoxine) or usual care only. Results showed a medium-sized, significant between-group effect on the YGTSS-TTS (in favor of CBIT plus usual care; *d*=0.56). The CBIT plus usual care-group further improved in a within-group analysis at a 3-month follow-up. This study provides preliminary evidence for CBIT being efficacious also in half of the previously evaluated dose. A final example of a shortened CBIT-protocol is the previously reported study by Bennett et al [21], where the treatment was shortened from 8 to 6 sessions for their very young sample (5–8 years), and still was shown to be efficacious. Another example of a more intensive treatment approach is the previously mentioned *“Tackle your tics”*-programme [53]. Further studies are needed to evaluate the intensity, spacing and duration of treatment sessions on the efficiency and effectiveness of BT.

## Survey on the use of psychological interventions among TS health care providers

As part of these European clinical guidelines, between October and November 2019, the ESSTS working group conducted a survey among 59 ESSTS members and TS experts. Compared to a previous similar survey conducted in 2011, the current survey showed that the popularity of psychological interventions increased over the course of eight years. In 2011, 47% of experts considered BT as a first-line intervention. In the current survey, the experts’ preference for BT as a first-line intervention increased to 63% (in the case of adults) and 79% (in the case of children). In 2011, no difference was made between children and adults. For medication, the opposite trend was observed. In 2011, 35% of the experts considered medication a first-line intervention, which dropped to 12% in adults and 5% in children in the 2019 survey. In the current survey, 80% of the experts stated that BT was available in their region for children, 59% stated that it was available for adults, while 15% stated it was unavailable. While that number seems relatively high, it would be wrong to imply that the supply met the demand in those regions. According to the experts’ estimations, only about 52% of the patients who were recommended BT actually had access to it. This resembles the findings of the 2011 survey, where 20 out of 40 respondents (50%) reported having difficulties in finding a knowledgeable provider for BT. Various modalities of treatment delivery were available in routine clinical care in the respondents’ regions, primarily individual face-to-face treatment (80%), and to a lesser extent internet-delivered treatment (22%), and group-delivered treatment (19%). Equivalent data were not available in the 2011 survey.

## Recommendations

Clinical consensus follows that psychoeducation is essential to help the patient and his/her environment to understand the condition and make well-informed treatment decisions. Psychoeducation is therefore recommended as the initial intervention for all individuals who are diagnosed with TS. Psychoeducation can be delivered without specialist training in psychotherapy. It should be individualized and meet the needs of the individual patient and his/her family. In cases where psychoeducation is judged to be a sufficient intervention for the patient, it is appropriate to adopt a *watch and wait* approach.

When psychoeducation alone is insufficient, BT (more specifically HRT/CBIT and ERP) is recommended as a first-line intervention for children and adults with tic disorders. Of the two BT interventions, HRT/CBIT has the strongest evidence-base. In the 2011 European clinical guidelines, several RCTs were reported which showed HRT/CBIT to be superior to various control conditions, of which the Piacentini et al. [8] pediatric trial currently is the largest study (*N* = 126). Since then, one major RCT (*N* = 122) has been published, showing that HRT/CBIT also is an effective treatment for adults [15]. Since 2011, no new RCTs have evaluated the use of ERP as a treatment for patients with TS. Based on one RCT [9], ERP is recommended as a treatment for patients with tic disorders, but at a lower certainty than HRT/CBIT due to considerably fewer published studies. To date, there is no appropriate evidence-base to make a differential indication as to when to apply HRT or ERP in particular. Verdellen et al. [9] report that “at face value” patients with a higher number of tics as assessed via the YGTSS dimension “Number of tics” could benefit more from ERP, since this method allows for a simultaneous treatment of multiple tics. Van de Griendt et al. [64] discussed from a theoretical point of view, that in the case of no tics and urges being present nor evocable during a therapy session, HRT could still be practiced and explained to the patient, while ERP could not properly be conveyed. Another theoretical point is that ERP could be given a preference in patients with comorbid obsessive–compulsive disorder (OCD) since ERP is the primary treatment for OCD [65]. In summary, there are no evidence-based indications as to when HRT or ERP is indicated. Clearly, future studies are necessary to provide an indication of what intervention works best for whom. For detailed information on how to deliver HRT/CBIT or ERP, including information on session amount and duration, see the published treatment manuals (e.g. [12, 13]).

Due to a current lack of controlled studies, cognitive interventions and third-wave interventions are not recommended as stand-alone treatments for patients with TS. However, it could be reasonable to offer such treatments as second-line interventions (or augmentations), if HRT/ERP has shown insufficient results and other evidence-based treatments (such as PT) are not available/possible or preferred by the patient. Notably, third-wave interventions have been shown to be effective treatments for other conditions, which are often co-occuring with tic disorders [66]. ACT has been shown to be effective for depression, anxiety, addiction and psychosomatic problems [67]. An improvement in any of these comorbid conditions could potentially, indirectly, contribute to an improvement of tic severity.

No new studies have been published on the efficacy of relaxation techniques (RT) since 2011. Because of a lack of sufficiently powered controlled studies the recommendation of RT is limited to a second-line intervention. An overview of the evidence on the efficacy of RT can be found here [68].

Since the 2011 European clinical guidelines were published, new treatment formats have been evaluated for the delivery of BT. At the moment, most evidence is found for videoconference delivery, which has been evaluated in two small RCTs [54, 55]. Additionally, there are some pilot data supporting the formats of group delivery and internet delivery, but more studies are warranted to enable firm recommendations.

## Current knowledge gaps and future directions

HRT/CBIT s is the psychological intervention for patients with TS that has the broadest evidence-base. A limitation of previously published trials of HRT/CBIT is that analyses on long-term durability have been limited to treatment responders and completer data [8, 15]. Due to tic disorders’ natural waxing and waning course, it is especially important to conduct well-controlled studies with long-term follow-up, using an intention-to-treat approach. For ERP to be recommended with the same certainty as HRT/CBIT, large RCTs in which ERP is compared to appropriate control conditions are warranted.

Regardless of the documented positive effects of BT, there is still room for improvement in the efficacy and efficiency of treatment delivery. In the two largest trials of BT (HRT/CBIT), reported between-group effect sizes were within the medium range (0.57 and 0.68) [8, 15]. Tic severity was reduced by 26–31% (as measured by the YGTSS-TTS), implicating that patients may still experience severe tics after being treated with BT. One way to make treatments more effective and efficient is to gain a better understanding of the underlying working mechanisms of BT. While studies of habituation as a working mechanism of BT for tics are equivocal [69, 70], other potential mechanisms should be empirically examined, e.g. urge tolerance, disconfirmation of beliefs that unpleasant urges cannot be tolerated, and increased inhibitory control [71, 72]. Enhancing the efficacy and efficiency of BT could also be done by studying predictors and moderators of treatment response, ways to increase treatment adherence, or the effects of booster sessions. Furthermore, it is unclear if and how the working mechanisms of HRT and ERP differ from each other, and whether combined approaches (such as studied by Nissen et al. [43]) or sequential/add-on approaches (such as studied by Verdellen et al. using a cross-over design [9]) offer additional benefits. Further, the added value of the generic interventions originating from functional analysis and/or relaxation as used in CBIT, needs to be addressed. Related to this, psychoeducation is also included in BT protocols, as well as being recommended as an initial intervention (prior to BT), but has not yet been evaluated in its own right, for example against a waitlist. From a theoretical perspective, a further understanding of the underlying learning processes and neurobiological correlates in TS treatment might be beneficial for improving BT for patients with TS.

The limited availability of BT for TS has led to an increased focus on dissemination and adaptation of treatment delivery in recent years. The ESSTS survey indicated that BT is fairly widely available now in the regions of ESSTS members (although more available for children than adults), but caution is warranted when interpreting these results since data primarily originated from specialist clinics. To further overcome geographical barriers and the lack of trained therapists, remote delivery of BT might be a solution. Especially, in the light of the currently ongoing COVID-19 pandemic, all forms of videoconferencing and internet-delivered interventions are becoming more vital. To our knowledge, three ongoing RCTs are currently evaluating two different internet-delivered BT interventions: internet-delivered CBIT without therapist support (called ONLINE-TICS, which is being evaluated in Germany [73]), and therapist-supported internet-delivered ERP (called BIP TIC ERP, which is being evaluated in both the UK [74] and Sweden [75]. Another ongoing RCT is evaluating group delivery of BT and modifications of treatment intensity [76]. Time will tell, if any of these modalities will become evidence-based interventions for patients with tic disorders.

RCTs of BT and RCTs of PT show roughly comparable effect sizes for both treatments (e.g. 0.57–0.68 for CBIT/HRT compared to PST, and 0.45–0.79 for various compounds of PT compared to placebo) [11, 77]. The recommendation of BT as a first-line treatment is based on the fact that BT has shown fewer (and less severe) side effects and longer-lasting treatment effects than PT (which are expected to dissipate with drug discontinuation). However, to be more precise about the differences in effects, side effects and sustainability of effects, RCTs comparing BT to PT head-to-head are needed. Furthermore, studies are needed to determine which TS patient will most likely benefit from either treatment. A small-scale RCT comparing ERP with risperidone is currently being conducted in terms of short- and long-term efficacy, cost-effectiveness, side effects and dropout rates [78]. In addition, the (potentially additional) effect of combining BT and PT needs to be studied further.

Comorbidities are common among individuals with TS, which implies a strong likelihood that clinicians delivering treatment for TS will need to consider making treatment adaptations to accommodate for one or several comorbid psychiatric disorders. Currently, data is lacking on how psychological treatments for TS should be adapted. This area needs to be studied to generate recommendations regarding the treatment of TS when comorbid conditions are present.

## Conclusions

Based on clinical consensus, psychoeducation is recommended as an initial intervention for all individuals who are diagnosed with TS. A *watch and wait* approach could be reasonable for patients without functional impairment from their tics, also considering that many young people likely experience a natural decrease in tics over time. When psychoeducation is insufficient, BT (HRT/CBIT and ERP) is recommended as a first-line intervention for children and adults with TS, if available. When comorbid psychiatric conditions are present, clinicians must adopt a pragmatic approach to guide decision-making on treatment adaptation and prioritization of what symptoms should be treated first. If there are unsatisfactory effects from BT, switching from one behavioral intervention (HRT/CBIT or ERP) to another or switching to PT can be considered. Alternatively, BT could be augmented with PT. Clinicians should also be aware that not all patients are motivated to undergo BT, hence it is important to always take each patient’s preferences into consideration. A decision tree summarizing all treatments recommended by the European clinical guidelines for patients with TS is presented in an editorial in the current issue of this journal.

## Supplementary Information

Below is the link to the electronic supplementary material.

Supplementary file1 (PDF 61 KB)

## Data Availability

The search algorithm is provided in the Online Resource 1. No further data or materials were collected.
