# Supplementary material for: European clinical guidelines for Tourette syndrome and other tic disorders—version 2.0. Part II: psychological interventions
Source: Eur Child Adolesc Psychiatry. 2021 Jul 27;31(3):403–23. doi: 10.1007/s00787-021-01845-z (PMC8314030; doi:10.1007/s00787-021-01845-z)
Supplement: Supplementary file 1 — Supplementary file1 (PDF 61 KB) [file 787_2021_1845_MOESM1_ESM.pdf]

European clinical guidelines for Tourette syndrome and other tic disorders—version 2.0. Part II: psychological interventions

European Child & Adolescent Psychiatry

Per Andrén, Ewgeni Jakubovski, Tara L. Murphy, Katrin Woitecki, Zsanett Tarnok, Sharon Zimmerman-Brenner, Jolande van de Griendt, Nanette Mol Debes, Paula Viefhaus, Sally Robinson, Veit Roessner, Christos Ganos, Natalia Szejko, Kirsten Müller-Vahl, Danielle Cath, Andreas Hartmann, Cara Verdellen

Correspondence to: Per Andrén, Clinical psychologist, MSc; Karolinska Institutet, Department of Clinical Neuroscience Child and Adolescent Psychiatry Research Center, Gävlegatan 22, 113 30 Stockholm, Sweden; E-mail: per.andren@ki.se

Online Resource 1

This search strategy was used to identify relevant studies.

Ovid search strategy from June 6, 2019

#	Searches	Results
1	tic disorders/ or tourette syndrome/	8252
2	Telemedicine/	24269
3	1 and 2	5
4	from 3 keep 1	1
5	Psychotherapy, Group/	13591
6	psychotherapy/ or behavior therapy/ or cognitive behavioral therapy/ or "acceptance and commitment therapy"/ or mindfulness/	174547
7	habit reversal.ti,ab,kw.	596
8	Comprehensive Behavioral Intervention.ti,ab,kw.	97
9	"exposure and response prevention".ti,ab,kw.	1311
10	5 or 6	183025
11	7 or 8 or 9	1947
12	1 and 10	399
13	11 and 12	108
14	Attention Deficit Disorder with Hyperactivity/	50744
15	Autistic Disorder/	19472

16	Obsessive-Compulsive Disorder/	27116
17	Comorbidity/	129896
18	14 or 15 or 16 or 17	217267
19	1 and 18	2437
20	13 or 19	2520
21	tourette syndrome/	7213
22	telemedicine/ or online therapy/ or telepsychiatry/ or telepsychology/	26640
23	21 and 22	6
24	group psychotherapy/ or psychotherapy/	133639
25	behavior therapy/	40426
26	cognitive behavior therapy/ or "acceptance and commitment therapy"/	42994
27	24 or 25 or 26	207113
28	habit reversal.ti,ab,id.	617
29	Comprehensive Behavioral Intervention.ti,ab,id.	103
30	"exposure and response prevention".ti,ab,id.	1320
31	28 or 29 or 30	1978
32	21 and 27	355
33	31 and 32	108
34	attention deficit disorder with hyperactivity/	50744
35	autism spectrum disorders/	46708
36	obsessive compulsive disorder/	27116
37	comorbidity/	129896
38	34 or 35 or 36 or 37	242830
39	21 and 38	2079
40	4 or 13 or 20	2521
41	23 or 33 or 39	2162
42	4 use medall	1
43	13 use medall	61

44	20 use medall	1612
45	42 or 43 or 44	1613
46	23 use psyh	5
47	33 use psyh	66
48	39 use psyh	940
49	46 or 47 or 48	992
50	45 or 49	2605
51	limit 50 to yr="2017 -Current"	153
**52**	**remove duplicates from 51**	**132**
